# Further validation to support clinical translation of [^18^F]FTC-146 for imaging sigma-1 receptors

**DOI:** 10.1186/s13550-015-0122-2

**Published:** 2015-09-17

**Authors:** Bin Shen, Michelle L. James, Lauren Andrews, Christopher Lau, Stephanie Chen, Mikael Palner, Zheng Miao, Natasha C. Arksey, Adam J. Shuhendler, Shawn Scatliffe, Kota Kaneshige, Stanley M. Parsons, Christopher R. McCurdy, Ahmad Salehi, Sanjiv S. Gambhir, Frederick T. Chin

**Affiliations:** Molecular Imaging Program at Stanford (MIPS) Department of Radiology Stanford University, Stanford, CA 94305 USA; Department of Neurology and Neurological Sciences, Stanford University, Stanford, CA 94305 USA; Department of Chemistry and Biochemistry, University of California Santa Barbara, Santa Barbara, CA 93106 USA; Department of Medicinal Chemistry and Pharmacology, The University of Mississippi, University, MS 38677 USA; Veterans Administration Palo Alto Health Care System, Palo Alto, CA 94304 USA

**Keywords:** Small animal PET, Sigma-1 receptor, [^18^F]FTC-146, Sigma-1 receptor knockout mice, Vesicular acetylcholine transporter, Dosimetry, Toxicology

## Abstract

**Background:**

This study aims to further evaluate the specificity and selectivity of [^18^F]FTC-146 and obtain additional data to support its clinical translation.

**Methods:**

The binding of [^19^F]FTC-146 to vesicular acetylcholine transporter (VAChT) was evaluated using [^3^H]vesamicol and PC12^A123.7^ cells in an in vitro binding assay. The uptake and kinetics of [^18^F]FTC-146 in S1R-knockout mice (S1R-KO) compared to wild-type (WT) littermates was assessed using dynamic positron emission tomography (PET) imaging. Ex vivo autoradiography and histology were conducted using a separate cohort of S1R-KO/WT mice, and radiation dosimetry was calculated from WT mouse data (extrapolated for human dosing). Toxicity studies in Sprague–Dawley rats were performed with a dose equivalent to 250× the anticipated clinical dose of [^19^F]FTC-146 mass.

**Results and discussion:**

VAChT binding assay results verified that [^19^F]FTC-146 displays negligible affinity for VAChT (*K*_i_ = 450 ± 80 nM) compared to S1R. PET images demonstrated significantly higher tracer uptake in WT vs. S1R-KO brain (4.57 ± 1.07 vs. 1.34 ± 0.4 %ID/g at 20–25 min, *n* = 4, *p* < 0.05). In S1R-KO mice, it was shown that rapid brain uptake and clearance 10 min post-injection, which are consistent with previous S1R-blocking studies in mice. Three- to fourfold higher tracer uptake was observed in WT relative to S1R-KO mouse brains by ex vivo autoradiography. S1R staining coincided well with the autoradiographic data in all examined brain regions (*r*^2^ = 0.85–0.95). Biodistribution results further demonstrated high [^18^F]FTC-146 accumulation in WT relative to KO mouse brain and provided quantitative information concerning tracer uptake in S1R-rich organs (e.g., heart, lung, pancreas) for WT mice vs. age-matched S1R-KO mice. The maximum allowed dose per scan in humans as extrapolated from mouse dosimetry was 33.19 mCi (1228.03 MBq). No significant toxicity was observed even at a 250X dose of the maximum carrier mass [^19^F]FTC-146 expected to be injected for human studies.

**Conclusions:**

Together, these data indicate that [^18^F]FTC-146 binds specifically to S1Rs and is a highly promising radiotracer ready for clinical translation to investigate S1R-related diseases.

**Electronic supplementary material:**

The online version of this article (doi:10.1186/s13550-015-0122-2) contains supplementary material, which is available to authorized users.

## Background

Based on sigma receptor (SR) binding studies, two subtypes have been characterized: sigma-1 receptors (S1Rs) and sigma-2 receptors (S2Rs) [1]. S1Rs are involved in a large range of basic biological processes and multiple chronic disease states, including neuropathic pain, depression, Alzheimer’s disease (AD), and schizophrenia [2–5]. S1R is a 26.2 kDa ligand-gated membrane chaperone protein that is Ca^2+^-sensitive, residing in the mitochondria-associated endoplasmic reticulum (ER) membrane, and is highly conserved across mammalian species [6]. Due to its subcellular localization, S1R is proposed to function as an interorganelle-signaling modulator [7]. Although some S1R agonists have showed efficacy as therapeutic agents in various neuropsychiatric diseases [8], vascular diseases [9], and cancer [10], the exact biochemical role of S1Rs in these disease states is yet to be fully elucidated.

During the last decade, a variety of small molecules have been developed for S1R-PET imaging [11]. Among those ligands, most human studies have been performed with [^11^C]SA4503 (S1R *K*_i_ = 4.6 nM) [12]. Although [^11^C]SA4503 has provided valuable information about S1R distribution in healthy controls, AD, and Parkinson’s disease patients [13], this radiotracer also displays significant affinity for S2R and the vesicular acetylcholine transporters (VAChT) (i.e., S2R *K*_i_ = 63 nM [9], VAChT *K*_i_ = 50 nM [14]; S2R/S1R = 13.6; VAChT/S1R = 10.9), confounding interpretation of its PET imaging data with respect to S1R. There were also other promising S1R radioligands, such as [^18^F]FPS [15] and [^18^F]fluspidine [16]. However, the former has only ~30-fold selectively for S1R compared to S2R receptors (Table 1) [17, 18], and neither of these imaging agents have been evaluated in S1R-KO mice.

**Table 1:** Comparison of sigma-1 receptor ligand binding affinities to S1R, S2R, and VAChT

						
	FTC-146	(−)-Vesamicol	SN-56	SA4503	FPS	Fluspidine
S1R *K*_1_ (nM)	0.0025	74 [24]	0.56	4.6	4.3 [17]	0.59 [18]
S2R *K*_1_ (nM)	364	364 [24]	>1000	63	142 [17]	708 [18]
VAChT *K*_1_ (nM)	450	20	140	50	N/A	1400 [18]

Ideally, a radiotracer should possess high specificity and selectivity for the target of interest in order to obtain specific and highly quantifiable imaging data. The specific and selective imaging of S1Rs with PET may provide critical insight about the in vivo distribution, expression levels, and functional role of these receptors in normal and aberrant physiology. We previously reported the synthesis and initial evaluation of an S1R antagonist radioligand, [^18^F]FTC-146 [19, 20]. In those studies, we showed that this tracer has picomolar affinity (S1R *K*_i_ = 2.5 pM) and a >1000-fold selectivity for S1R over S2R. Mouse PET imaging corroborated the specific binding of [^18^F]FTC-146 to S1Rs in vivo through blocking studies with known S1R ligands. Moreover, we observed specific uptake of [^18^F]FTC-146 in S1R-rich regions of rat and in non-human primate brain. While all of these data are encouraging, the affinity of our tracer for VAChT, a common off-target binding site for S1R ligands, was not evaluated. Additionally, while we have shown that [^19^F]FTC-146 does not display significant affinity for 59 other central nervous system (CNS) targets by in vitro binding studies, the absolute specificity and selectivity of this tracer for S1Rs in vivo has not been determined.

In the current study, we set out to determine the specificity and selectivity of [^18^F]FTC-146 for S1Rs, validating its use for specific PET imaging of S1R levels. Furthermore, we sought to accumulate other information, including dosimetry and toxicology, required for the translation of this PET tracer to the clinic. The amalgamation of our current findings regarding the application of [^18^F]FTC-146 to WT and age-matched S1R-KO mice with our previous findings provides definitive evidence that our tracer is highly specific and selective for S1Rs, both in vitro and in vivo. To the best of our knowledge, this is the first study to evaluate a S1R radiotracer in S1R-KO mice and to demonstrate a correlation between tracer accumulation and S1R immunostaining in brain.

## Methods

### General

Unless otherwise stated, chemicals were purchased from commercial sources and used without further purification. BD1047 for mice studies was purchased from Sigma Aldrich. [^19^F]FTC-146 (99.1 % chemical purity) reference compound was provided by Albany Molecular Research Inc. (Albany, NY). Radiochemistry and semi-preparative high performance liquid chromatography (HPLC) was performed using a TRACERlab FX-FN module (GE Healthcare) with an ancillary HPLC pump (Dionex P680) and UV detector (KNAUER K-2001). Analytical HPLC was performed on a quaternary pump (Agilent 1200 series) equipped with an autosampler, a photodiode array UV detector, and a model 105S single-channel radiation detector (Carroll & Ramsey Associates). Radiotracer doses for animal studies were measured using a dose calibrator (Capintec, CRC-15 PET). Positron emission tomography/computed tomography (PET/CT) imaging of mice was performed using microPET/CT hybrid scanner (Siemens Inveon). All PET data were reconstructed using a 3-dimensional ordered subsets expectation maximization algorithm (16 subsets, 4 iterations) with scatter and dead time correction, and a matrix size of 128 × 128 × 159. Attenuation correction was applied to dataset from CT image.

### VAChT binding affinity

In vitro binding assays for VAChT were conducted with human VAChT stably expressed in PC12^A123.7^ cells at about 50 pmol/mg of crude extract. No significant amounts of S1Rs and S2Rs were present. The VAChT radioligand used was 5 nM (−)-[^3^H]vesamicol (24.3 Ci/mmol; 899 GBq/mmol), and the assay was conducted at final concentrations of 10^−11^ M to 10^−4^ or 10^−5^ M of S1R-targeting compounds [21]. Nonspecific binding was determined with 5 μM (±)-vesamicol. Unlabeled (−)-vesamicol was used as an external standard (*K*_i_ ~ 15 nM) and the sealed mixtures were allowed to equilibrate at 23 °C for 20 h. Duplicate data were averaged and fitted by regression of a rectangular hyperbola to estimate the *K*_i_ values of the novel compounds (Additional file 1: Figure S1).

### Animals

Oprs1 mutant (+/−) OprsGt (IRESBetageo) 33Lex litters on a C57BL/6 J × 129 s/SvEv mixed background were purchased from the Mutant Mouse Regional Resource Center, UC Davis, CA, from which homozygous WT (Sigma-1 receptor +/+) and S1R-KO (Sigma-1 receptor −/−) mice were obtained through in-house breeding. All mice were maintained on a 4 % fat diet (Harland Teklad, 8604 M/R) and subjected to standard light cycles (12 h/12 h light–dark). Stanford University Institutional Animal Care and Use Committee approved all experimental procedures involving animals.

### Genotyping

Mice were bred onsite, and tail samples were collected from mice litters and sent to Transnetyx Inc to determine genotype (Cordova, TN, USA). Mice were always re-genotyped at the conclusion of each experiment.

### Radiochemistry

[^18^F]FTC-146 was synthesized via aliphatic nucleophilic substitution (^18^F/tosylate exchange) using TRACERlab FX-FN as previously described [19]. Briefly, tosylate precursor solution (2 mg in 1 mL anhydrous DMSO) was added into azeotropically dried ^18^F/K_222_/K_2_CO_3_ complex, heated to 150 °C for 15 min, and then the crude product was purified on semi-prep HPLC. The [^18^F]FTC-146 HPLC fraction was formulated in saline containing no more than 10 % ethanol.

### Small animal magnetic resonance imaging (MRI)

Mice were scanned with a dedicated small animal 7 Tesla Varian Magnex Scientific MR scanner with custom-designed pulse sequences and radiofrequency (RF) coils using standard methods. The scanner consisted of a superconducting magnet (Magnex Scientific) with 7.0 Tesla field strength, a gradient insert (Resonance Research, Inc.) with clear bore size of 9 cm (770 mT/m, 2500 T/m/s), a General Electric (GE) console, and Copley 266 amplifiers. For each MR scan, mice were anesthetized using isoflurane gas (2.0–3.0 % for induction and 1.5–2.5 % for maintenance). Body temperature was continually monitored and maintained within the normal range using a temperature sensor. Respiration rate and oxygen saturation were also monitored throughout scan. Coronal brain images were acquired using T2-weighted fast spin echo sequences (TE/TR 58.5 ms/4000 ms) using 9 NEX, a 256 × 256 matrix, 3 cm field of view, slice thickness of 500 μm, and a total imaging time of 19 min. Mouse brain MRI data was used to provide an anatomical reference frame for PET/CT imaging.

### Small animal PET/CT imaging

Mice were anesthetized using isoflurane inhalation (2.0–3.0 % for induction and 1.5–2.5 % for maintenance, isofluorane in oxygen at a flow rate of 2 L/min). Following tail vein cannulation, each catheterized mouse was placed in a custom-made 4 × 4 mouse bed. All mice were kept warm using an infrared warming pad (Kent Scientific) placed under and around the bed while under isoflurane anesthesia in the microPET/CT scanner (pixel size 200 μm). A 5-min CT scan was performed just prior to each PET scan. Acquisition of dynamic PET data (4 × 15 s, 4 × 60 s, 11 × 300 s) for WT (*n* = 4) and S1R-KO (*n* = 4) mice commenced just prior to intravenous administration of [^18^F]FTC-146 (45–130 μCi; 1.67–4.81 MBq), followed by 50 μL sterile heparinized saline to flush cannula, and yielded a total of 19 frames over 60 min. Blocking studies involved pre-treating WT (*n* = 4) and S1R-KO (*n* = 4) mice with BD1047 (1 mg/kg, Sigma Aldrich) 10 min prior to radioligand administration. PET, CT, and MR images were imported into Inveon Research Workspace 4.0 (Siemens) and co-registered using the automatic affine registration. Tracer activity over time was measured in regions of interest in the PET image (mean activity in the region) by using each brain MR image as an anatomical guide (Additional file 2: Figure S2). Regions of interest were selected prior to the commencement of this study, and included previously reported S1R-rich brain structures (i.e., caudate putamen, cerebellum, cortex, and hippocampus) [22].

### Ex vivo biodistribution and stability

WT (*n* = 5) and S1R-KO mice (*n* = 5) were administered [^18^F]FTC-146 (60–90 μCi; 2.2-3.3 MBq) via tail vein injection. Blood samples were collected via cardiac puncture 25 min following tracer administration, and each mouse was perfused with saline (10–20 mL) prior to sacrificing and removing organs of interest (i.e., brain, heart, kidney, liver, lung, muscle, pancreas, small intestine, spleen, stomach). The radioactivity in weighed organs was assessed in automated gamma counter (Cobra II; Packard) and decay corrected to the time of radiotracer injection using diluted aliquots of the initial administered dose as standards. Another set of WT mice (*n* = 4) were used to evaluate the ex vivo stability of [^18^F]FTC-146 in brain, liver, kidney, small intestine, and stomach homogenates at 25 min after tracer administration (using previously reported methods [20]).

### Ex vivo autoradiography

Coronal brain sections from WT (*n* = 3), heterozygous (*n* = 1), and S1R-KO (*n* = 4) mice were harvested 30 min post-injection of [^18^F]FTC-146 (200–300 μCi; 7.4–11.1 MBq). After perfusing each mouse with saline (15–20 mL), the brain was removed and divided into two sagittal halves. Half of each brain was embedded in optimal cutting temperature (OCT) compound (Tissue-Tek) and frozen on dry ice for ex vivo autoradiography, while the other half was fixed in 4 % paraformaldehyde (PFA) for immunohistochemistry. Muscle samples from front leg, known to contain negligible levels of S1R, were also dissected and embedded alongside corresponding brain tissue to be used as an internal reference region for each mouse in order to normalize ex vivo autoradiography data. Coronal brain and muscle sections (20 μm thick) were cut using a cryostat microtome HM500 (Microm), mounted on microscope slides (Fisherbrand SuperfrostTM Plus Microscope Slides), air-dried for at least 10 min, and then exposed to ^18^F-sensitive storage phosphor screens (Perkin Elmer) overnight at −20 °C. Each phosphor screen was scanned using a Typhoon 9410 Variable Mode Imager (Amersham Biosciences) and image data were visualized and quantified using ImageJ (Image processing and analysis software in Java, version 1.45 s). Anatomy of brain sections was confirmed by Nissl staining (cresyl violet acetate; Sigma Aldrich) using standard techniques.

### Immunohistochemistry

S1R immunostaining was performed using free-floating 40-μm-thick coronal brain sections from WT (*n* = 3) and S1R-KO (*n* = 4) mice. Sections were incubated in a 1 % H_2_O_2_, 50 % TBS (pH = 7.4)/MeOH solution for 30 min to quench the endogenous peroxidase activity and subsequently blocked with 10 % normal goat serum (NGS; Vector Laboratories) in TBST (1 % Triton X-100, Sigma Aldrich) for 1 h to reduce nonspecific staining and to permeabilize the tissue. Sections were then incubated with S1R specific primary antibody (1:400, [23]) containing 5 % NGS and TBST overnight at room temperature. Following this, the sections were incubated with biotinylated anti-rabbit secondary antibody 1:200 (Vector Laboratories) in 5 % NGS and TBST for 1 h at room temperature and then with avidin-biotin complex (diluted 1:1000 in TBS, Vector Laboratories) for 90 min at room temperature. Finally, sections were incubated with 3,3′-diaminobenzidine (DAB; Sigma Aldrich) for 10 min, mounted on slides, dehydrated, and then cover-slipped with Permount (Sigma Aldrich). Images of the immunostained sections were taken with a Nanozoomer (Hamamatsu), and images were quantified using ImageJ.

### Dosimetry

Mice (WT, female *n =* 3; male *n =* 3) were imaged for 2 h using Inveon PET/CT following a high-resolution CT (pixel size 103.2 μm). An additional two 10-min static PET/CT scans at 4 and 6 h were performed on these mice that were allowed to be awake between scans. ROIs were drawn over the brain, thyroid, heart, liver, lung, gallbladder, spleen, kidneys, urinary bladder, bone, muscle, and testicle/uterus of the mice by using the image analysis software IRW (Siemens) with the help of the CT images. The %ID/g associated with each ROI was extracted and underwent an animal-human biokinetic extrapolation using the percent kg/g method. Source organ residence times were calculated using a standard quantitation platform organ level internal dose assessment (OLINDA) utilizing a bi-exponential model without bladder voidance. The projected human radiation doses were then computed for male and female phantoms using the source organ residence times.

### Toxicity of [^19^F]FTC-146

This study was conducted by SoBran Bioscience (Baltimore, MD). It consisted of one test article treatment group of ten male and ten female Sprague–Dawley rats dosed with [^19^F]FTC-146 at 0.069 mg/kg, equivalent to 250× the anticipated clinical dose mass. An additional group of ten males and ten females received the vehicle, 10 % ethanol in sterile saline, and served as the control. All rats received a dose volume of 5 mL/kg. The rats were dosed intravenously once on study day 1. Five male and five female rats from each group were bled on study day 3 and the remaining rats were bled on study day 15. All animals were euthanized and necropsied following blood collection. Parameters evaluated for test article effect included survival, clinical observations, body weight, body weight gain, clinical pathology, gross pathology, organ weights, and microscopic pathology.

### Statistical analyses

Results are presented as mean value ± standard deviation (STDV) for radiochemistry, PET/CT imaging, ex vivo biodistribution and stability and dosimetry studies; mean value ± standard error of the mean (SEM) is shown for ex vivo autoradiography and immunohistochemistry studies. Unpaired *t* tests (one tail) were used to compare PET/autoradiography/IHC data from WT with S1R-KO mice. Correlations between autoradiography mean signal intensity and S1R staining were determined by Pearson correlation test. All statistical analyses were performed using Microsoft Excel (version 14.0.7) or GraphPad Prism software (version 6.0c; GraphPad). A *p* value of less than 0.05 was considered as statistically significant.

## Results

### Radiochemistry

[^18^F]FTC-146 was obtained with radiochemical yield of 2.8 ± 1.2 % and specific radioactivity of 4.1 ± 2.1 Ci/μmol (151.7 ± 77.7 GBq/μmol). Both radiochemical and chemical purities were >99 %. All radiochemical yields and specific radioactivities were decay corrected to end of bombardment (*n* = 11).

### VAChT binding affinity

Competitive inhibition experiments using (−)-[^3^H]vesamicol were performed to estimate affinities of FTC-146 and SN-56 for VAChT (Table 1). Unlabeled (−)-vesamicol was used as an internal standard and was found to have an affinity of *K*_i_ = 13 ± 1 nM for VAChT, which is within the typical range known for this compound [24]. In the same assay, FTC-146 was shown to have an affinity of *K*_i_ = 450 ± 80 nM for VAChT, which is 180,000-fold less affinity than its picomolar affinity for S1R [19]. The non-fluorinated S1R compound, SN-56, was found to have an affinity of *K*_i_ = 140 ± 18 nM for VAChT in the same assay.

### PET/CT/MR imaging

**Fig. 1:**
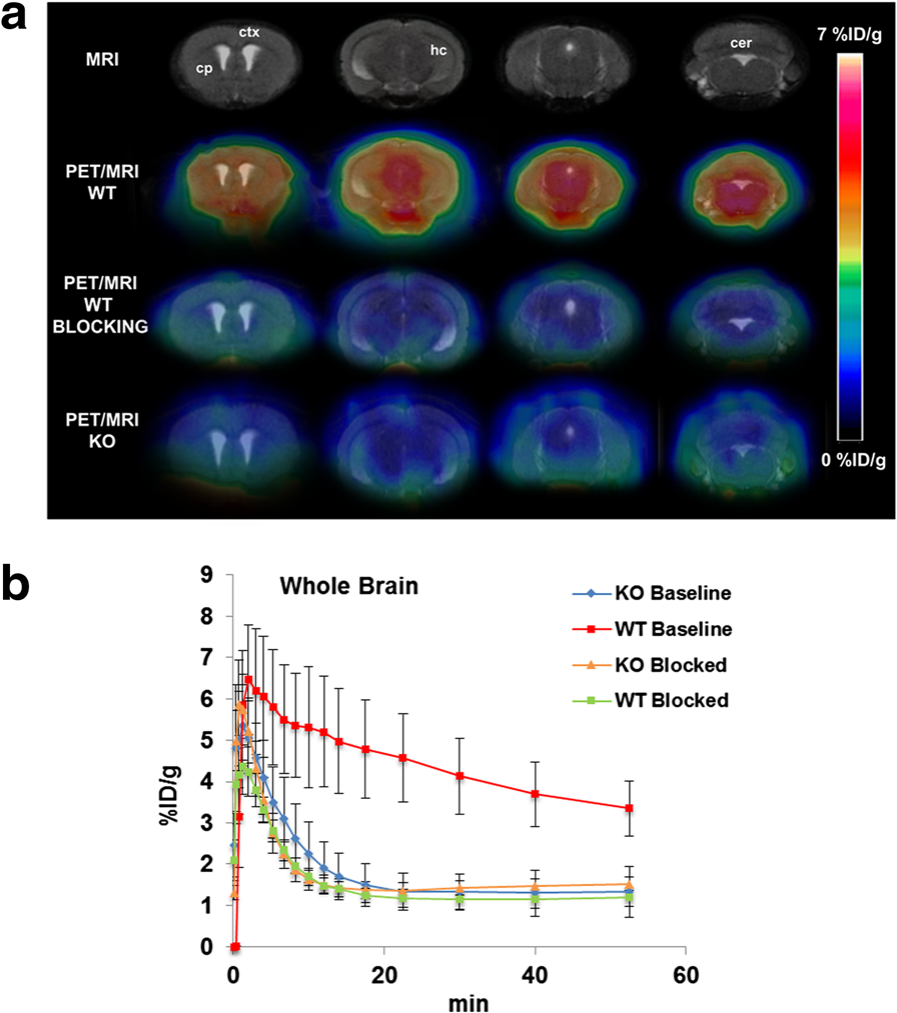
**a** Representative coronal PET/MR brain images of baseline WT/S1R-KO and blocking WT groups (*n* = 4 per group; blocking with BD1047 at 1 mg/kg). *ctx* = cortex; *cp* = caudate putamen; *hc* = hippocampus; *cer* = cerebellum. **b** Time activity curves depicting accumulation of [^18^F]FTC-146 in whole brain over time. We observed significantly higher brain uptake in baseline WT mice compared to S1R-KO mice at all time points during 60 min PET scan (*P* < 0.05). The kinetics of [^18^F]FTC-146 in WT blocking, S1R-KO baseline, and S1R-KO blocking studies all exhibited similar kinetics: i.e., the tracer quickly entered and cleared from the brain within 10 min post-injection

**Fig. 2:**
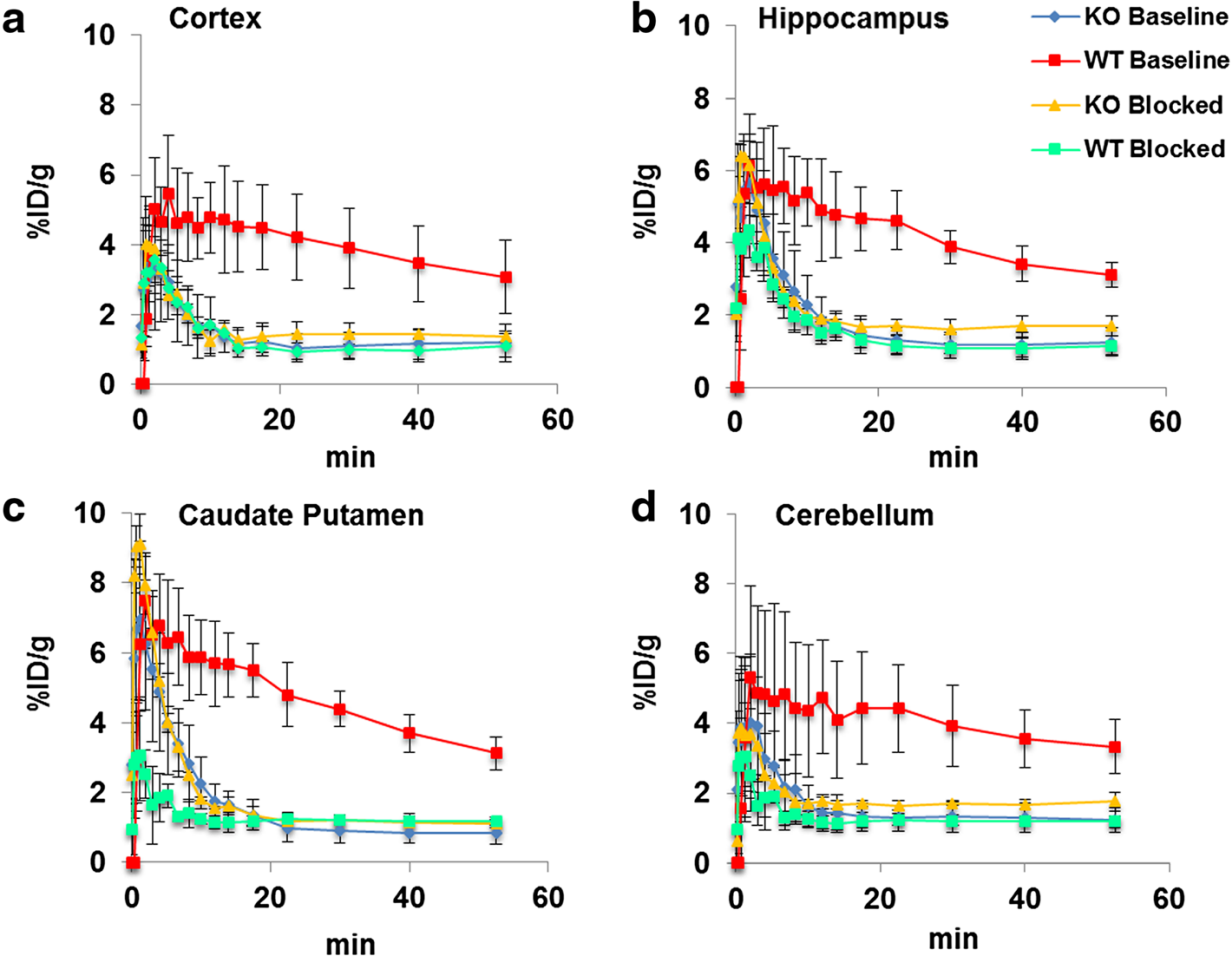
Time activity curves depicting accumulation of [^18^F]FTC-146 in **a** cortex, **b** caudate putamen, **c** hippocampus, and **d** cerebellum for baseline and blocking studies in WT and S1R-KO mice (*n* = 4 per group; blocked with BD1047 at 1 mg/kg)

In vivo [^18^F]FTC-146 uptake and pharmacokinetics in WT and S1R-KO mice were assessed by dynamic PET/CT imaging. Representative whole brain PET/MR images and time activity curves for both WT and S1R-KO mice are shown in Fig. 1. These graphs demonstrated that [^18^F]FTC-146 entered the brain of both WT and S1R-KO mice rapidly with peak uptake occurring within 3 min post-injection (6.46 ± 1.33 %ID/g for WT; 5.07 ± 0.88 %ID/g for S1R-KO, *n* = 4), with much faster washout observed in S1R-KO mice compared to WT mice (76 % reduction vs. 37 % reduction at 30 min post-injection). Overall, we found significantly higher accumulation of [^18^F]FTC-146 in whole brain of WT mice compared to S1R-KO mice (whole brain: 4.57 ± 1.07 vs. 1.34 ± 0.44 %ID/g at 20 min, *n* = 4, *p* < 0.05). Similarly, in several S1R-rich brain regions (Fig. 2), we observed marked accumulation of [^18^F]FTC-146 in WT baseline studies, while S1R-KO brains, as well as WT and S1R-KO brains following receptor blocking with a known S1R antagonist (BD-1047), showed similarly rapid washout of tracer.

### Ex vivo biodistribution and stability

**Fig. 3:**
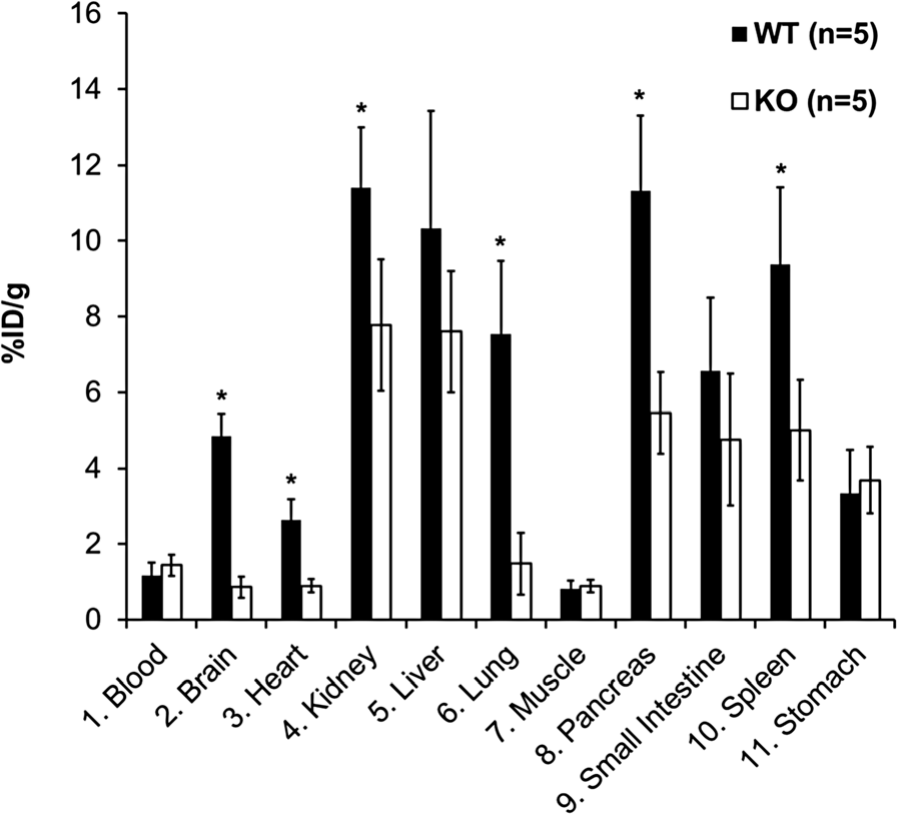
Ex vivo biodistribution of [^18^F]FTC-146 in mice 25 min after injection of radioligand (*n =* 5, *p* < 0.05)

To quantitatively determine the uptake of [^18^F]FTC-146 in WT versus S1R-KO mice, we performed ex vivo biodistribution studies 25 min post-injection of tracer (Fig. 3). Similar to our PET imaging results, we observed significantly higher uptake of [^18^F]FTC-146 in the brain of WT mice compared to S1R-KOs (biodistribution: 4.85 ± 0.59 vs. 0.86 ± 0.28 %ID/g, *p* < 0.05; PET: 4.57 ± 1.07 vs. 1.34 ± 0.4 %ID/g at 25 min, *p* < 0.05). In addition, we observed significantly greater [^18^F]FTC-146 uptake in peripheral organs known to contain moderate to high levels of S1Rs [6, 25], including the lung (7.54 ± 1.93 vs. 1.48 ± 0.83 %ID/g, *p* < 0.05), heart (2.63 ± 0.55 vs. 0.90 ± 0.17 %ID/g, *p* < 0.05), pancreas (11.32 ± 1.98 vs. 5.46 ± 1.08 %ID/g, *p* < 0.05), spleen (9.37 ± 2.06 vs. 5.00 ± 1.33 %ID/g, *p* < 0.05), and kidney (11.40 ± 1.58 vs. 7.78 ± 1.74 %ID/g, *p* < 0.05). However, we did not find significant differences between WT and S1R-KO [^18^F]FTC-146 uptake in muscle, whole blood, small intestine, or stomach, in which muscle and blood contain very low if any S1Rs; and small intestine and stomach contain notable levels of S1Rs [26, 27]. Lastly, our ex vivo [^18^F]FTC-146 stability results showed 100 % intact [^18^F]FTC-146 in the brain, but different levels of degradation in liver (29 % intact), stomach (45 % intact), small intestine (55 % intact), and kidney (34 % intact) (Additional file 3: Table S1).

### Ex vivo autoradiography

**Fig. 4:**
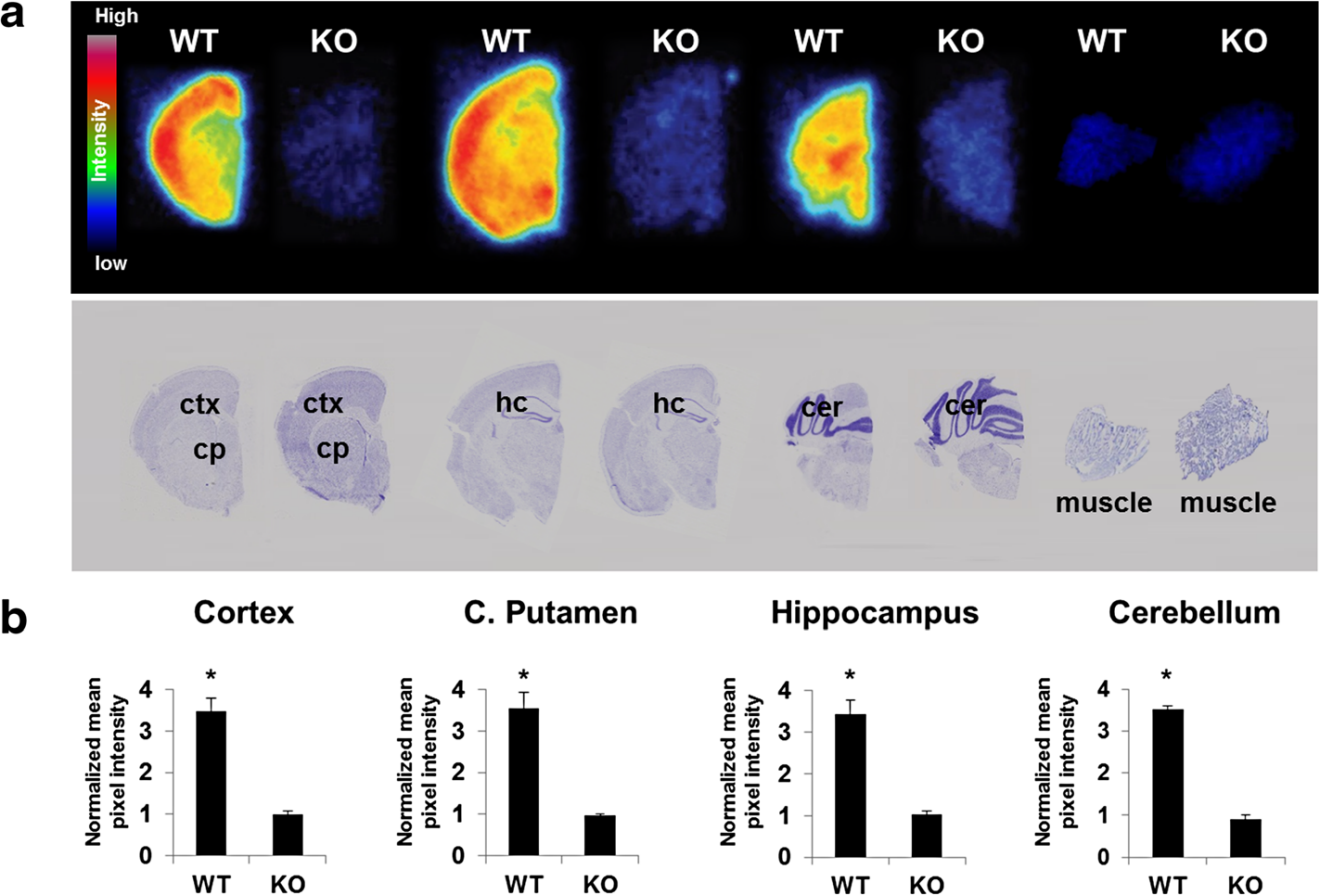
Ex vivo autoradiography of S1R-KO and WT mice (25 min P.I.). **a** Top row contains representative autoradiography images of [^18^F]FTC-146 from coronal sections throughout the brain of WT & S1R-KO (KO) mice. Second row shows Nissl staining of same sections used for autoradiography (for anatomical comparison). **b** Mean signal intensity for specific brain regions normalized to muscle for WT and KO mice. We observed three- to fourfold higher uptake of [^18^F]FTC-146 in all analyzed brain regions for WT mice compared to KO mice (*n* = 3 WT; *n* = 4 S1R-KO)

The distribution of [^18^F]FTC-146 in mouse brain was further evaluated by high-resolution ex vivo autoradiography. In WT mice, [^18^F]FTC-146 mainly accumulated in the cortex, cerebellum, caudate putamen, and hippocampus. Similar to in vivo PET results, there was significantly less [^18^F]FTC-146 uptake in the brain of S1R-KO mice compared to WT mice. The level of tracer accumulation in all regions of S1R-KO mouse brain was comparable with that observed in muscle. The normalized autoradiography signals in all WT brain regions analyzed (WT vs. S1R-KO) are as follows: 3.53 ± 0.07 vs. 0.91 ± 0.10 in cortex; 3.54 ± 0.39 vs. 0.97 ± 0.03 in caudate putamen; 3.44 ± 0.33 vs. 1.03 ± 0.08 in hippocampus; 3.47 ± 0.33 vs. 1.01 ± 0.06 in cerebellum (*n* = 3 for WT, *n* = 4 for S1R-KO; *p* < 0.05, Fig. 4).

### Immunohistochemistry

**Fig. 5:**
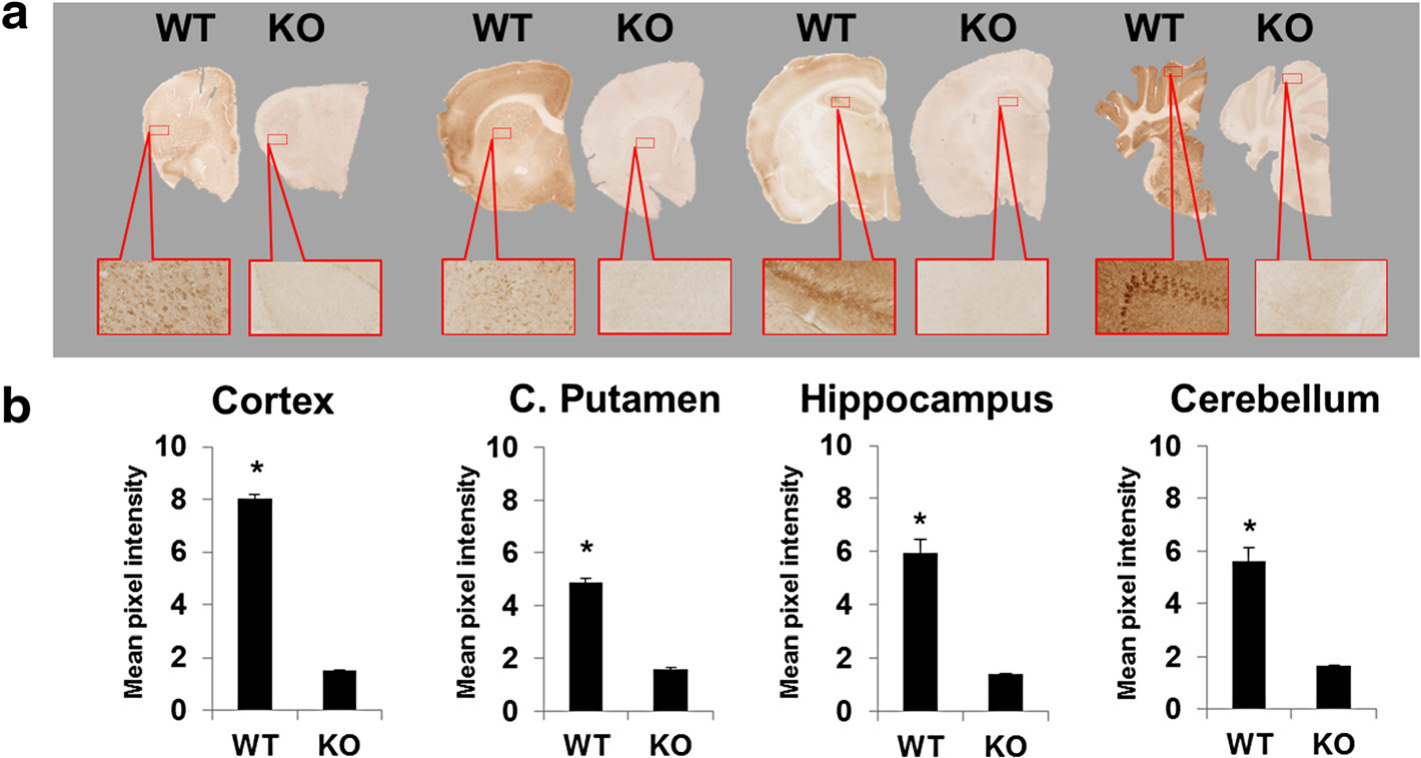
**a** Representative coronal S1R-stained WT and S1R-KO mouse brain sections. Images were all acquired using a nanozoomer with the same settings and are all shown on the same scale (40× magnified). **b** Staining differences between WT and S1R-KO brain sections were calculated by comparing pixel intensities of regions of interest after normalizing the sections to their background reading. (*n* = 3 WT; *n* = 4 S1R-KO)

**Fig. 6:**
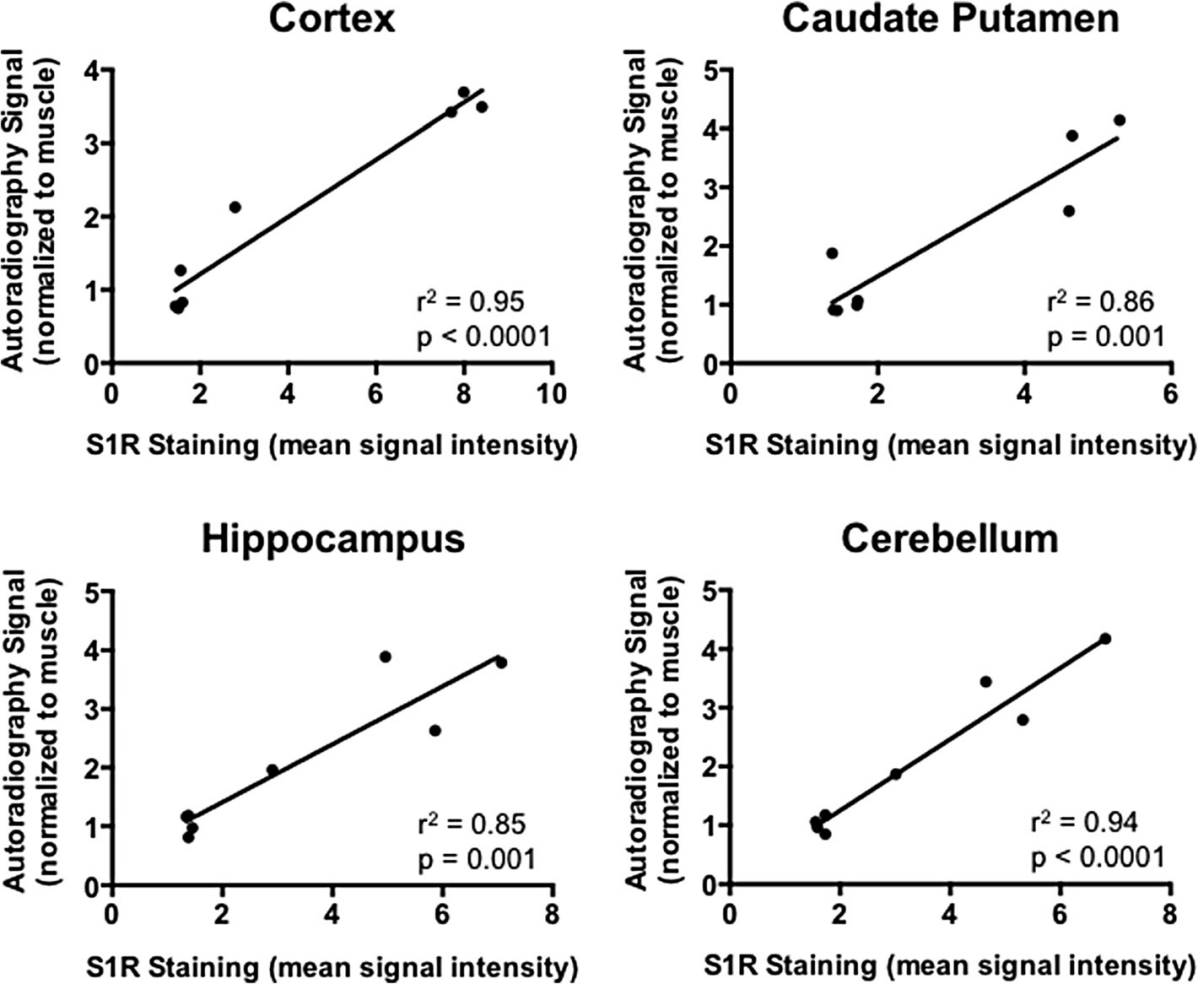
Correlation between autoradiography signal and S1R immunostaining for the cerebellum, cortex, caudate putamen, and hippocampus of S1R-KO (−/−), heterozygous (+/−), and WT mice (*n* = 3 WT; *n* = 1 heterozygous; *n* = 4 S1R-KO)

In order to evaluate the relationship between [^18^F]FTC-146 autoradiography signal and levels of S1R staining, we conducted S1R-immunohistochemistry with brain tissue from the same mice used for autoradiography studies. Results from these studies revealed high levels of S1R staining in brain tissue from WT mice, especially in the cortex, caudate putamen, hippocampus, and cerebellum. S1R-KO mouse brain sections were found to be devoid of S1R staining. Mean pixel intensities for WT vs. S1R-KO brain sections were found to be 7.99 ± 0.16 vs. 1.53 ± 0.03 for cortex; 5.29 ± 0.18 vs. 1.57 ± 0.07 for caudate putamen; 4.96 ± 0.50 vs. 1.39 ± 0.02 for hippocampus; and 6.82 ± 0.52 vs. 1.65 ± 0.04 for cerebellum (*n* = 3 for WT, *n* = 4 for S1R-KO, *p* < 0.05, Fig. 5). Autoradiography and S1R staining were also performed on brain tissue from a S1R-KO heterozygous (+/−) mouse in order to investigate correlation between S1R staining levels and autoradiography signal. A strong correlation between S1R expression level and autoradiography signal was found in the cerebellum (*r*^2^ = 0.94) and cortex (*r*^2^ = 0.95), whereas there was a moderate to strong correlation in the hippocampus (*r*^2^ = 0.85) and caudate putamen (*r*^2^ = 0.86) (Fig. 6).

### Dosimetry

A representative whole body 3D image [^18^F]FTC-146 in WT mice and region of interest is presented in Additional file 4: Figure S3. The estimated radiation doses as extracted from mice were 0.032 rem/mCi (1.18 rem/MBq) in females and 0.011 rem/mCi (0.41 rem/MBq) in males. The estimated absorbed radiation doses to human target organs are presented in Additional file 5: Table S2. Highest radiation doses were found in the spleen, urinary bladder, and bone. The dose-limiting organ was the bone (osteogenic cells), since bone is considered a critical organ for active blood-formation with a limit of 3 rem/single dose (5 rem/yearly). The single/yearly dose limit to human female or male was estimated to 33.19 mCi (1228.03 MBq) single dose/55.31 mCi (2046.47 MBq) yearly dose and 50.34 mCi (1862.42 MBq) single dose/83.89 mCi (3104.03 MBq) yearly dose, respectively.

### Toxicity of [^19^F]FTC-146

Following treatment with FTC-146, animals remained bright, alert, and responsive at all times and did not exhibit signs of toxicity during the conduct of the study. No treatment-related differences were noted in mean body weights, clinical chemistry, hematology, or coagulation parameters. In addition, no treatment-related effects were observed for organ weights or in gross and microscopic pathology. A brief summary of this data is presented in Additional file 6: Table S3.

## Discussion

S1Rs have received much attention from the molecular imaging community due to their involvement in a variety of cancers and neurological disorders [28, 29]. Highly specific and selective S1R radioligands are critical to understanding the in vivo role of this receptor and how its levels change over time in these disease states. Such radioligands could enable noninvasive detection and staging of diseases, e.g., AD and chronic pain, as well as monitoring novel S1R-related therapeutics.

The aim of the current work was to further investigate the selectivity, specificity, dosimetry, and safety of a promising, previously reported S1R radioligand, [^18^F]FTC-146. We wanted to determine its utility to provide accurate in vivo images of S1R density and to provide a foundation to translate [^18^F]FTC-146 for clinical imaging.

The present work provided definitive evidence concerning the high specificity and selectivity of [^18^F]FTC-146 for S1Rs, both in vitro and in vivo*.* Specifically, we found that [^19^F]FTC-146 has only negligible in vitro affinity for VAChT compared to its picomolar S1R affinity (i.e., VAChT *K*_i_ = 450 nM, S1R *K*_i_ = 0.0025 nM) and that there is only negligible [^18^F]FTC-146 brain uptake in S1R-KO mice compared to age-matched WTs. We also showed that [^18^F]FTC-146 brain autoradiography results for WT and S1R-KO mice corresponded well with PET imaging results and that there is a good correlation between [^18^F]FTC-146 accumulation and extent of S1R immunostaining in all brain regions investigated (i.e., caudate putamen, cerebellum, cortex, and hippocampus).

This S1R-KO study also allowed to us to capture a global perspective of FTC-146 binding affinity to all brain targets including emopamil-binding protein (EBP), which is known to display significant sequence homology to the S1R protein and is thus another potential off-target binding site for S1R ligands [30]. For example, SA4503 has been reported to display low nanomolar affinity for EBP (*K*i = 1.7 nM). Since we observed negligible uptake of [^18^F]FTC-146 in S1R-KO mice compared to age-matched WTs, we considered this to be good validation of this tracer’s high selectivity/specificity for S1R in vivo, and confirmation that this tracer does not interact significantly with EBP in vivo.

Biodistribution data also illustrated significantly higher [^18^F]FTC-146 uptake in the brain and most S1R-rich peripheral organs in WTs compared with S1R-KO mice (between two- and fivefold). However, we did not observe a significant difference in the liver, stomach, or small intestine uptake, which have all previously been reported as S1R-rich organs [27]. This is likely due to the presence of radioactive metabolites produced in the liver and then subsequently passed to the stomach and small intestine (see Additional file 3: Table S1). In addition, there is a higher [^18^F]FTC-146 concentration in blood from S1R-KO mice compared to WTs, which is likely due to the lower amount of tracer binding in these mice (which lack S1Rs) leading to more tracer being available to circulate in blood. Finally, from mouse radiation dosimetry, we estimate that bone would be the rate-limiting organ with an estimated absorbed radiation dose in human males of 0.090 rem/mCi. Defluorination was observed in mice in previous study [19]. However [^18^F]FTC-146 showed more chemical stability when it was studied in larger species, such as rats and squirrel monkeys [20]. Therefore, we can assume that the bone uptake may be even lower once a full human dosimetry study has been carried out. Lastly, [^19^F]FTC-146 toxicity study in Sprague–Dawley rats proves the amount of [^19^F]FTC-146 included in a radiotracer dose likely is safe for human administration.

To the best of our knowledge, this is the first study to evaluate a S1R radiotracer in S1R-KO mice and to demonstrate a correlation between tracer uptake and S1R immunostaining in different mouse brain regions. These additional findings provide critical information to understand the selectivity/specificity of a radiotracer for its desired target and for determining what the PET/autoradiography imaging signal actually represents.

## Conclusions

The results achieved from this study, together with the fact that [^18^F]FTC-146 has the highest in vitro affinity and selectivity for S1R over S2R of any published radioligand to date, illustrate the potential of [^18^F]FTC-146 for serving as a clinically useful, highly accurate imaging tool for investigating S1Rs in health and disease. We are currently evaluating the utility of [^18^F]FTC-146 for detecting and monitoring S1R-related neurological disorders, including chronic pain and AD. Additionally, based on these encouraging findings, we are now moving forward with an exploratory Investigational New Drug application, utilizing the dosimetry estimation in WT mice and evaluation of tracer toxicity we have performed, for approval from the U.S. Food and Drug Administration to allow us to perform our first-in-human tracer biodistribution and dosimetry studies with clinical-grade [^18^F]FTC-146.

## Ethical approval

Stanford University Institutional Animal Care and Use Committee approved all experimental procedures involving animals.

## Informed consent

This article does not contain any studies with human participants performed by any of the authors.

## Additional files

Additional file 1: Figure S1. FTC-146, SN-56 and Vesamicol binding affinity to VAChT. (DOC 171 kb) Additional file 2: Figure S2. Representative MR images showing how regions of interest (ROIs) were drawn for 1 = cortex; 2 = caudate putamen; 3 = hippocampus, 4 = cerebellum during PET/MR image analysis. Whole brain ROIs were drawn using the skull from the CT image as a guide. (DOC 573 kb) Additional file 3: Table S1.
*Ex vivo* stability of [^18^F]FTC-146 in WT mouse (*n =* 4). (DOC 29 kb) Additional file 4: Figure S3. Representative microPET/CT 3D summed image from 30–45 min post injection of [^18^F]FTC-146 (Left). Region of interest (ROI) drawn with the help of CT anatomical images (Right). (DOC 505 kb) Additional file 5: Table S2. Estimated human radiation doses extrapolated from mouse data. (DOC 40 kb) Additional file 6: Table S3. Summary of [^19^F]FTC-146 toxicity study in rats. (DOC 34 kb)
