# Supplementary figures and images for: Further validation to support clinical translation of [18F]FTC-146 for imaging sigma-1 receptors
Source: EJNMMI Res. 2015 Sep 17;5:49. doi: 10.1186/s13550-015-0122-2 (PMC4573970; doi:10.1186/s13550-015-0122-2)

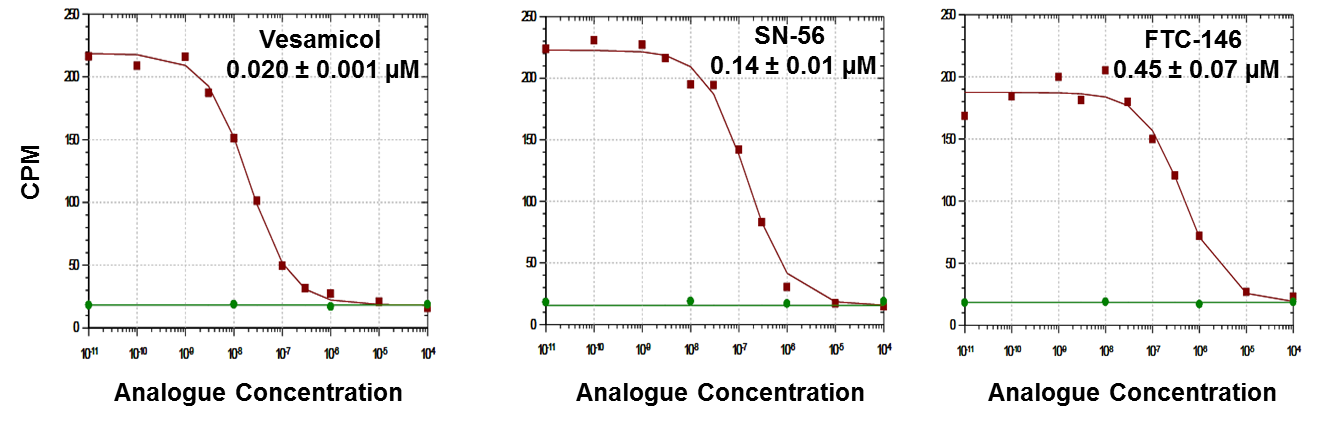


**Supplementary Fig. S1.** FTC-146, SN-56 and Vesamicol binding affinity to VAChT.

Supplement: Additional file 1: Figure S1. — FTC-146, SN-56 and Vesamicol binding affinity to VAChT. (DOC 171 kb) [file 13550_2015_122_MOESM1_ESM.doc]
