# Supplementary material for: Further validation to support clinical translation of [18F]FTC-146 for imaging sigma-1 receptors
Source: EJNMMI Res. 2015 Sep 17;5:49. doi: 10.1186/s13550-015-0122-2 (PMC4573970; doi:10.1186/s13550-015-0122-2)
Supplement: Additional file 2: Figure S2. — Representative MR images showing how regions of interest (ROIs) were drawn for 1 = cortex; 2 = caudate putamen; 3 = hippocampus, 4 = cerebellum during PET/MR image analysis. Whole brain ROIs were drawn using the skull from the CT image as a guide. (DOC 573 kb) [file 13550_2015_122_MOESM2_ESM.doc]

**Supplementary Fig. S2.** Representative MR images showing how regions of interest (ROIs) were drawn for 1 = cortex; 2 = caudate putamen; 3 = hippocampus, 4 = cerebellum during PET/MR image analysis. Whole brain ROIs were drawn using the skull from the CT image as a guide.


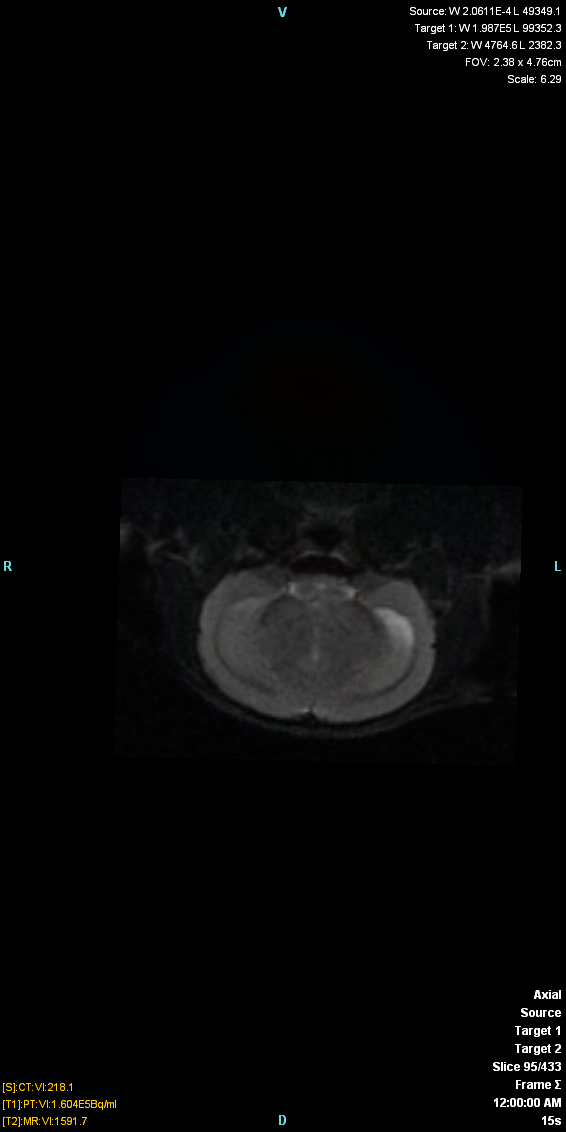

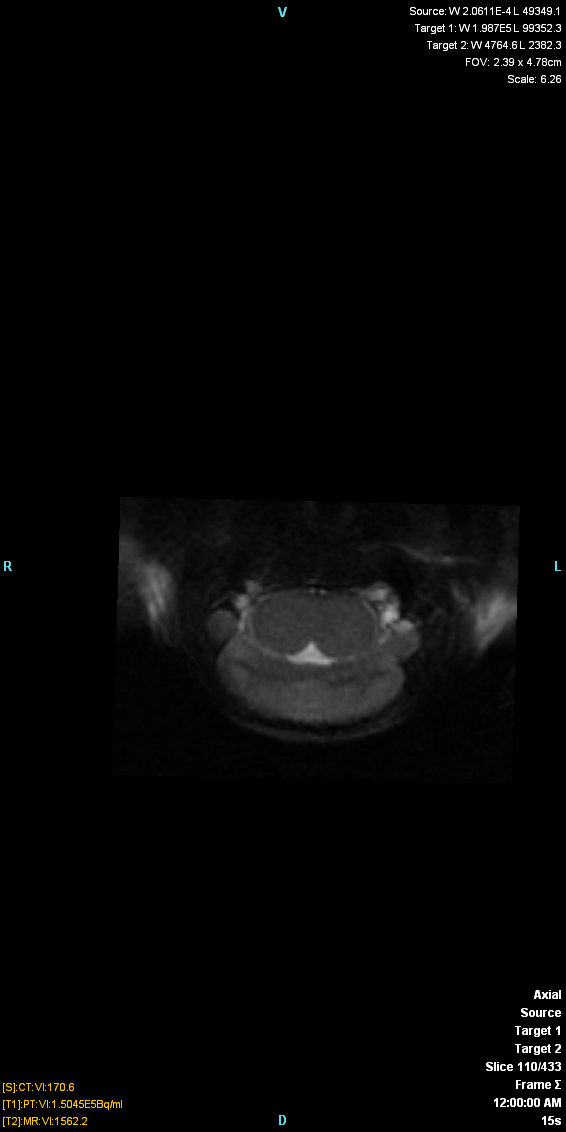

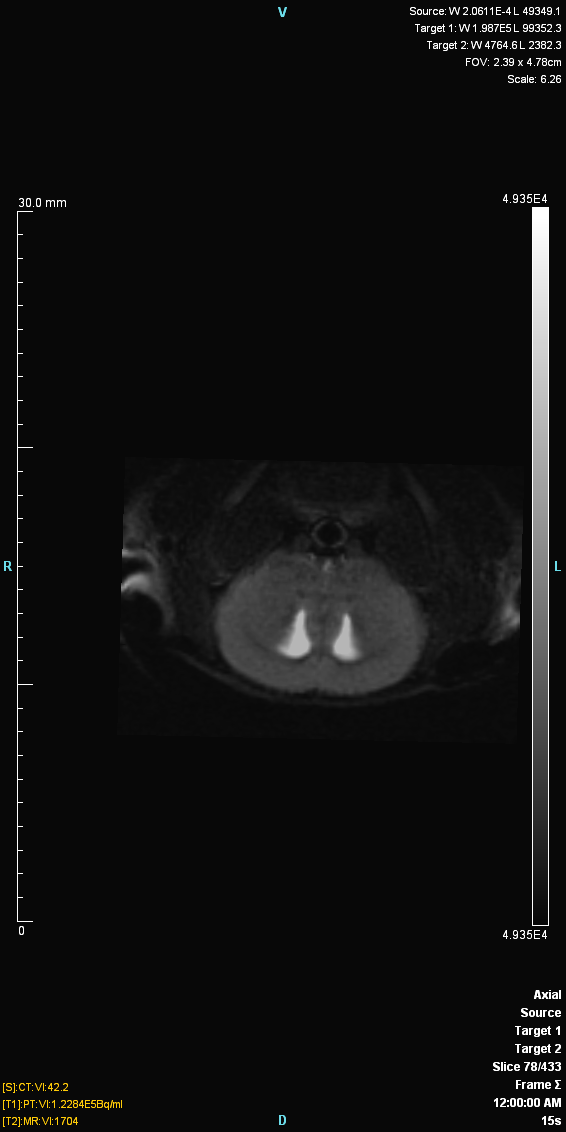


**1**

**2**

**3**

**4**
