# Supplementary material for: Further validation to support clinical translation of [18F]FTC-146 for imaging sigma-1 receptors
Source: EJNMMI Res. 2015 Sep 17;5:49. doi: 10.1186/s13550-015-0122-2 (PMC4573970; doi:10.1186/s13550-015-0122-2)
Supplement: Additional file 3: Table S1. — Ex vivo stability of [18F]FTC-146 in WT mouse (n = 4). (DOC 29 kb) [file 13550_2015_122_MOESM3_ESM.doc]

**Supplementary Table. S1**. *Ex vivo* stability of [^18^F]FTC-146 in WT mouse (*n =* 4).

|  | Small Intestine | Liver | Stomach | Kidney | Brain |
| --- | --- | --- | --- | --- | --- |
| Intact [^18^F]FTC-146 (%) ^1^ | 54.7 ± 7.8 | 28.8 ± 8.4 | 44.6 ± 8.3 | 34.0 ± 10.5 | 100 ± 0 |
| Radioactivity extraction efficiency (%) ^2^ | 73.5 ± 7.8 | 56.1 ± 7.4 | 65.8 ± 8.3 | 68.9 ± 10.7 | 87.1 ± 2.4 |

***^1^****: The percent ratio of intact [^18^F]FTC-146 to the total radioactivity on the HPLC chromatogram*

***^2^****: Samples (100 μL) from each supernatant were measured in a gamma counter to assess the extraction efficiency into supernatant liquids*
