# Supplementary material for: Further validation to support clinical translation of [18F]FTC-146 for imaging sigma-1 receptors
Source: EJNMMI Res. 2015 Sep 17;5:49. doi: 10.1186/s13550-015-0122-2 (PMC4573970; doi:10.1186/s13550-015-0122-2)
Supplement: Additional file 4: Figure S3. — Representative microPET/CT 3D summed image from 30–45 min post injection of [18F]FTC-146 (Left). Region of interest (ROI) drawn with the help of CT anatomical images (Right). (DOC 505 kb) [file 13550_2015_122_MOESM4_ESM.doc]

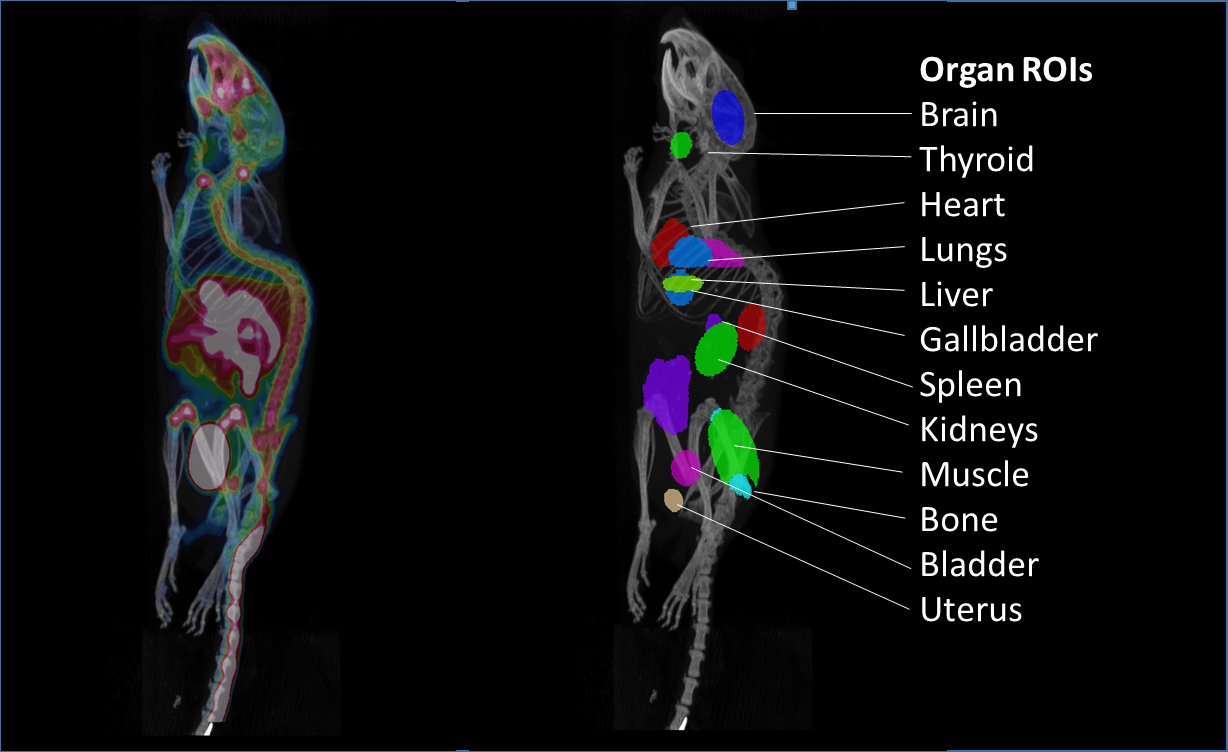


**Supplementary Fig. S3.** Representative microPET/CT 3D summed image from 30-45 minutes post injection of [^18^F]FTC-146 (Left). Region of interest (ROI) drawn with the help of CT anatomical images (Right).
