# Supplementary material for: Further validation to support clinical translation of [18F]FTC-146 for imaging sigma-1 receptors
Source: EJNMMI Res. 2015 Sep 17;5:49. doi: 10.1186/s13550-015-0122-2 (PMC4573970; doi:10.1186/s13550-015-0122-2)
Supplement: Additional file 5: Table S2. — Estimated human radiation doses extrapolated from mouse data. (DOC 40 kb) [file 13550_2015_122_MOESM5_ESM.doc]

**Supplementary Table. S2.** Estimated human radiation doses extrapolated from mouse data.

| **Organ** | Human total dose equivalent | | | |
| --- | --- | --- | --- | --- |
|  | **Female** | | **Male** | |
|  | rem/mCi | rem/MBq | rem/mCi | rem/MBq |
| Adrenals | 3.60E-02 | 1.33 | 2.75E-02 | 1.02 |
| Brain | 3.83E-02 | 1.42 | 2.90E-02 | 1.07 |
| Breasts | 2.18E-02 | 0.81 | 1.80E-02 | 0.66 |
| Gallbladder Wall | 4.58E-02 | 1.69 | 3.41E-02 | 1.26 |
| Heart Wall | 3.30E-02 | 1.22 | 2.77E-02 | 1.02 |
| Kidneys | 4.36E-02 | 1.61 | 3.55E-02 | 1.31 |
| Liver | 7.03E-02 | 2.60 | 4.58E-02 | 1.69 |
| Lungs | 2.63E-02 | 0.97 | 2.09E-02 | 0.77 |
| Muscle | 2.85E-02 | 1.05 | 2.44E-02 | 0.90 |
| Ovaries | 3.21E-02 | 1.19 | NR | NR |
| Pancreas | 3.60E-02 | 1.33 | 3.00E-02 | 1.11 |
| Red Marrow | 4.67E-02 | 1.73 | 3.02E-02 | 1.12 |
| Osteogenic Cells | **9.04E-02** | **3.34** | **5.96E-02** | **2.21** |
| Skin | 2.03E-02 | 0.75 | 1.64E-02 | 0.61 |
| Spleen | 7.23E-02 | 2.68 | 7.88E-02 | 2.92 |
| Testes | NR | NR | 2.22E-02 | 0.82 |
| Thyroid | 5.25E-02 | 1.94 | 3.16E-02 | 1.17 |
| Urinary Bladder Wall | 2.51E-02 | 0.93 | 2.64E-02 | 0.98 |
| Uterus | 3.20E-02 | 1.18 | NR | NR |
| Effective Dose | 4.06E-02 | 1.50 | 3.20E-02 | 1.18 |
