# Supplementary material for: Further validation to support clinical translation of [18F]FTC-146 for imaging sigma-1 receptors
Source: EJNMMI Res. 2015 Sep 17;5:49. doi: 10.1186/s13550-015-0122-2 (PMC4573970; doi:10.1186/s13550-015-0122-2)
Supplement: Additional file 6: Table S3. — Summary of [19F]FTC-146 toxicity study in rats. (DOC 34 kb) [file 13550_2015_122_MOESM6_ESM.doc]

**Supplementary Table. S3.** Summary of [^19^F]FTC-146 toxicity study in rats.

|  | Treatment | Body Weights | Clinical Observations | Clinical Pathology | Organ Weights | Pathology/Histology |
| --- | --- | --- | --- | --- | --- | --- |
| Day 3 | Treated with saline | **NSC** | **NAF** | **NAF** | **NSC** | **NAF** |
|  | Treated with [^19^F]FTC-146 | **NSC** | **NAF** | **Hematology:** **NAF**  **Coagulation**: **NAF**  **Chemistry: 40 %** increase in Total Bilirubin (TBIL); **40 %** decrease in Triglycerides (TRIG) for Females | **NSC** | **NAF** |
| Day 15 | Treated with saline | **NSC** | **NAF** | **NAF** | **NSC** | **NAF** |
|  | Treated with [^19^F]FTC-146 | **NSC** | **NAF** | **Hematology:** **15.9 %** increase in reticulocyte counts for Males  **Coagulation:** **NAF**  **Chemistry**: **34.9 %** increase in Alanine Aminotransferase (ALT) for Males; **13.6 %** decrease in Cholesterol (CHOL) for Females | **2.6 %** increase in liver and **17.8 %** increase in spleen weights in Males | **NAF** |

***NSC:*** *No significant changes.*

***NAF:*** *No abnormal findings.*

***Note:*** *Data showing statistical significance is not believed to be biologically meaningful because of the small magnitude of the difference from the control values.*
